# Survey of prothioconazole sensitivity in *Fusarium pseudograminearum* isolates from Henan Province, China, and characterization of resistant laboratory mutants

**DOI:** 10.1186/s12870-023-04714-w

**Published:** 2024-01-04

**Authors:** Feng Zhou, Yan Jiao, Aohui Han, Xiaoli Zhou, Jiamei Kong, Haiyan Hu, Runqiang Liu, Chengwei Li

**Affiliations:** 1 Postdoctoral Research Base, Henan Institute of Science and Technology, Xinxiang, 453003 China; 2 Henan Engineering Research Center of Crop Genome Editing , Henan International Joint Laboratory of Plant Genetic Improvement and Soil Remediation, Henan Institute of Science and Technology, Xinxiang, 453003 China; 3 School of Food Science and Engineering, Henan University of Technology, Zhengzhou, 450001 China; 4 Henan Engineering Research Center of Green Pesticide Creation and Pesticide Residue Monitoring By Intelligent Sensor, Henan Institute of Science and Technology, Xinxiang, 453003 China

**Keywords:** *Fusarium pseudograminearum*, Prothioconazole, Fungicide resistance, Resistance mechanism, Cross-resistance

## Abstract

**Background:**

Fusarium crown rot (FCR) is one of the most significant diseases limiting crop production in the Huanghuai wheat-growing region of China. Prothioconazole, a triazole sterol 14α-demethylation inhibitor (DMI) fungicide developed by the Bayer Crop Protection Company, is mainly registered for the prevention and control of wheat powdery mildew and stripe rust (China Pesticide Information Network). It is known to exhibit high activity against *F. pseudograminearum*, but further research, particularly regarding the potential for fungicide resistance, is required before it can be registered for the control of FCR in China.

**Results:**

The current study found that the baseline sensitivity of 67 field isolates of *F. pseudograminearum* collected between 2019 and 2021 ranged between 0.016–2.974 μg/mL, with an average EC_50_ value of 1.191 ± 0.720 μg/mL (mean ± SD). Although none of the field isolates exhibited signs of resistance, three highly resistant mutants were produced by repeated exposure to prothioconazole under laboratory conditions. All of the mutants were found to exhibit significantly reduced growth rates on potato dextrose agar (PDA), as well as reduced levels of sporulation, which indicated that there was a fitness cost associated with the resistance. However, inoculation of wounded wheat coleoptiles revealed that the pathogenicity of the resistant mutants was little affected or actually increased. Molecular analysis of the genes corresponding to the prothioconazole target protein, FpCYP51 (*FpCYP51A, FpCYP51B,* and *FpCYP51C*), indicated that the resistant mutants contained three conserved substitutions (M63I, A205S, and I246V) that were present in the *FpCYP51C* sequence of all three mutants, as well as several non-conserved substations in their *FpCYP51A* and *FpCYP51B* sequences. Expression analysis revealed that the presence of prothioconazole (0.1 μg/mL) generally resulted in reduced expression of the three *FpCYP51* genes, but that the three mutants exhibited more complex patterns of expression that differed in comparison to their parental isolates. The study found no evidence of cross-resistance between prothioconazole and any of the fungicides tested including three DMI fungicides tebuconazole, prochloraz, and flutriafol.

**Conclusions:**

Taken together these results not only provide new insight into the resistant mechanism and biological characteristics associated with prothioconazole resistance in *F. pseudograminearum*, but also strong evidence that prothioconazole could provide effective and sustained control of FCR, especially when applied in combination with other fungicides.

**Supplementary Information:**

The online version contains supplementary material available at 10.1186/s12870-023-04714-w.

## Background

Wheat is one of the most important cereal crops that sustains more than 1/3 of the world's population, and maintaining consistent production is therefore critical to global food security. It has been estimated that wheat cultivation in China covers a total area of 2.34 million hectares with an annual production of 13.43 million tons per year [1, 2]. However, recent research has found that infection with Fusarium crown rot (FCR) resulted in an average yield loss of 36,000 tons per year in Henan Province between 2017 and 2021 alone, with the highest annual loss being 44,000 tons [3]. Indeed, in recent years, FCR, which is primarily caused by *Fusarium pseudograminearum*, has emerged as one of the most destructive soil-borne diseases of wheat and other economically important cereal crops throughout the world [4, 5]. Furthermore, FCR infection, not only threatens crop yields, but also grain quality as mycotoxin contamination poses a danger to both human and livestock health [5]. It has also been noted that FCR has a long period of development before visible symptoms become evident, which makes the disease difficult to manage resulting in a high risk of substantial crop damage [5]. Consequently, FCR has been listed as a high priority disease for early warning and control in the Henan, Hebei, Shandong and Shaanxi Provinces of China [5].

It is generally recognized that the development of resistant varieties would be the most effective means to control FCR and prevent mycotoxin contamination of harvested grain [5]. However, in the absence of such varieties, the application of chemical fungicides can provide effective management of FCR [5], but as this disease has only recently emerged in some parts of China, there are currently no fungicides specifically registered for the control of FCR [5, 6]. Prothioconazole is a broad spectrum triazole fungicide developed by the Bayer Crop Protection Company in 2004. Previous research has shown that prothioconazole inhibits the 14α-demethylation of lanolin, which is the precursor of sterol biosynthesis in fungi, and therefore should be considered a member of the sterol 14α-demethylation inhibitor (DMI) group of fungicides [6–8]. In addition to its broad-spectrum activity, prothioconazole has many other desirable characteristics including good internal absorption, long duration of activity, and low environmental impact [9]. The Fungicide Resistance Action Committee (FRAC) classifies DMI fungicides in the G1 subgroup of Sterol Biosynthesis Inhibitors (SBI) that are recommended for general use in all crops [10], and prothioconazole is widely used for the prevention and control of disease in field crops such as wheat, soybean, and rice [6, 9]. Indeed, studies have shown that prothioconazole provides excellent control of almost all fungal diseases, with especially high activity against wheat scab, dry blight, and powdery mildew [6], and that when applied at a rate of 200 g/hm^2^ it provided equal to or better control than other commonly used fungicides such as epoxiconazole, tebuconazole and cyprodinil [9, 11].

Given the versatility and usefulness of prothioconazole, the potential for the emergence of fungicide resistance is of great concern. Previous studies have shown that the mechanism of resistance to other DMI fungicides is primarily caused by overexpression or mutations associated with the CYP51 target site, or overexpression of genes encoding drug efflux pumps [12–14]. The fungal CYP51 protein, which is also known as sterol 14α-demethylase, is a member of the cytochrome superfamily and its primary function is to remove the methyl group from the 14α-site of the sterol precursor. However, it has also been noted that most pathogenic fungi encode more than one 14α-demethylase gene [15–17]. For example, *Fusarium graminearum* contains at least three CYP51 homologs genes, including *FgCYP51A*, *FgCYP51B* and *FgCYP51C*. In addition, previous studies have shown that point mutations related to fungicide resistance mainly occur in the FgCYP51A and FgCYP51B homologs [18, 19]. The current study was initiated to investigate the potential for prothioconazole resistance in the closely related species *F. pseudograminearum*, and determine whether similar resistant mechanisms might also occur. The specific objectives of the study were to: (i) monitor field resistance in the Henan Province of China, and establish the baseline sensitivity for prothioconazole in *F. pseudograminearum*; (ii) evaluate the fitness parameters of resistant and sensitive isolates; (iii) determine the potential for cross-resistance between prothioconazole and other commonly used fungicides, including tebuconazole, prochloraz, carbendazim, flutriafol, pyraclostrobin, fluazinam, and fludioxonil, and (iv) investigate the molecular basis for prothioconazole resistance in *F. pseudograminearum*.

## Results

### The sensitivity determination of field isolates of *F. pseudograminearum* from Henan Province, China to prothioconazole

The current study found no evidence of prothioconazole resistance among 67 field isolates of *F. pseudograminearum* collected between 2019 and 2021, with none of the isolates being capable of growth on PDA amended with 5.0 µg/mL prothioconazole. Indeed, further investigation revealed that the isolates were sensitive to prothioconazole with EC_50_ values ranging from 0.016–2.974 μg/mL (Table 1; Fig. S1), and an average EC_50_ ± standard deviation (SD) of 1.191 ± 0.72 μg/mL.

**Table 1:** Prothioconazole sensitivity (EC_50_) of 67 *F. pseudograminearum* isolates collected from wheat fields in the Henan Province of China between 2019 and 2021

Isolates	Location	EC_50_ (μg/mL)	Year	Isolates	Location	EC_50_ (μg/mL)	Year
XC-1	Xuchang	0.933	2019	HNYY-2021–43-a	Xinxiang	0.869	2021
JZ-1	Jiaozuo	1.342	2019	HNYY-2021–46-a	Xinxiang	1.617	2021
SQ-1	Shangqiu	1.066	2019	HNYY-2021–50-a	Xinxiang	2.781	2021
NL-1	Shangqiu	0.776	2019	HNYY-2021–4-b	Xinxiang	1.954	2021
XX-1	Xiangxiang	1.126	2019	HNYY-2021–5-b	Xinxiang	2.214	2021
HB-1	Hebi	1.063	2019	HNYY-2021–7-b	Xinxiang	1.119	2021
ZK-1	Zhoukou	1.424	2019	HNYY-2021–8-b	Xinxiang	0.067	2021
SQ-3	Shangqiu	0.619	2019	HNYY-2021–9-b	Xinxiang	0.2	2021
ZK-2	Zhoukou	2.602	2019	HNYY-2021–11-b	Xinxiang	0.1	2021
YY-1	Zhengzhou	1.361	2019	HNYY-2021–12-b	Xinxiang	0.045	2021
HNXX-2020-YJ-1-c	Xinxiang	1.669	2020	HNYY-2021–13-b	Xinxiang	0.072	2021
HNXX-2020-YJ-2-c	Xinxiang	1.324	2020	HNYY-2021–19-b	Xinxiang	0.132	2021
HNXX-2020-YJ-3-c	Xinxiang	2.178	2020	HNYY-2021–20-b	Xinxiang	2.929	2021
HNXX-2020-YJ-4-c	Xinxiang	1.312	2020	HNYY-2021–21-b	Xinxiang	0.016	2021
HNXX-2020-YJ-5-c	Xinxiang	1.058	2020	HNYY-2021–22-b	Xinxiang	0.618	2021
XX-4	Zhengzhou	1.87	2020	HNYY-2021–25-b	Xinxiang	1.054	2021
HNYY-2020–15-a	Xinxiang	0.746	2020	HNYY-2021–29-b	Xinxiang	1.084	2021
HNYY-2020–23-a	Xinxiang	0.449	2020	HNYY-2021–32-b	Xinxiang	0.428	2021
HNYY-2020–28-a	Xinxiang	1.363	2020	HNYY-2021–34-b	Xinxiang	1.315	2021
HNYY-2020–30-a	Xinxiang	0.851	2020	HNYY-2021-36b	Xinxiang	2.230	2021
HNYY-2020–33-a	Xinxiang	0.328	2020	HNYY-2021–37-b	Xinxiang	1.979	2021
HNYY-2020–35-a	Xinxiang	0.24	2020	HNYY-2021–39-b	Xinxiang	0.821	2021
HNYY-2020–38-a	Xinxiang	0.441	2020	HNYY-2021–42-b	Xinxiang	1.618	2021
HNYY-2021–10-c	Xinxiang	1.289	2021	HNYY-2021–45-b	Xinxiang	1.618	2021
HNYY-2021–14-c	Xinxiang	0.851	2021	HNYY-2021–48-b	Xinxiang	1.651	2021
HNYY-2021–16-c	Xinxiang	1.830	2021	HNYY-2021–51-b	Xinxiang	1.38	2021
HNYY-2021–17-c	Xinxiang	0.382	2021	HNYY-2021–53-b	Xinxiang	1.474	2021
HNYY-2021–18-c	Xinxiang	0.179	2021	HNYY-2021–2-c	Xinxiang	1.359	2021
HNYY-2021–24-c	Xinxiang	2.218	2021	HNYY-2021–3-c	Xinxiang	1.344	2021
HNYY-2021–26-c	Xinxiang	1.003	2021	HNYY-2021–6-c	Xinxiang	1.979	2021
HNYY-2021–27-c	Xinxiang	0.85	2021	HNYY-2021–41-c	Xinxiang	0.821	2021
HNYY-2021–31-c	Xinxiang	1.849	2021	HNYY-2021–47-c	Xinxiang	2.974	2021
HNYY-2021–40-c	Xinxiang	1.307	2021	HNYY-2021–49-c	Xinxiang	1.052	2021
HNYY-2021–52-c	Xinxiang	1.011	2021	/	/	/	/

/ Indicates no data available

### Mycelial growth, sporulation and pathogenicity of three prothioconazole-resistant mutants of *F. pseudograminearum*

**Fig. 1:**
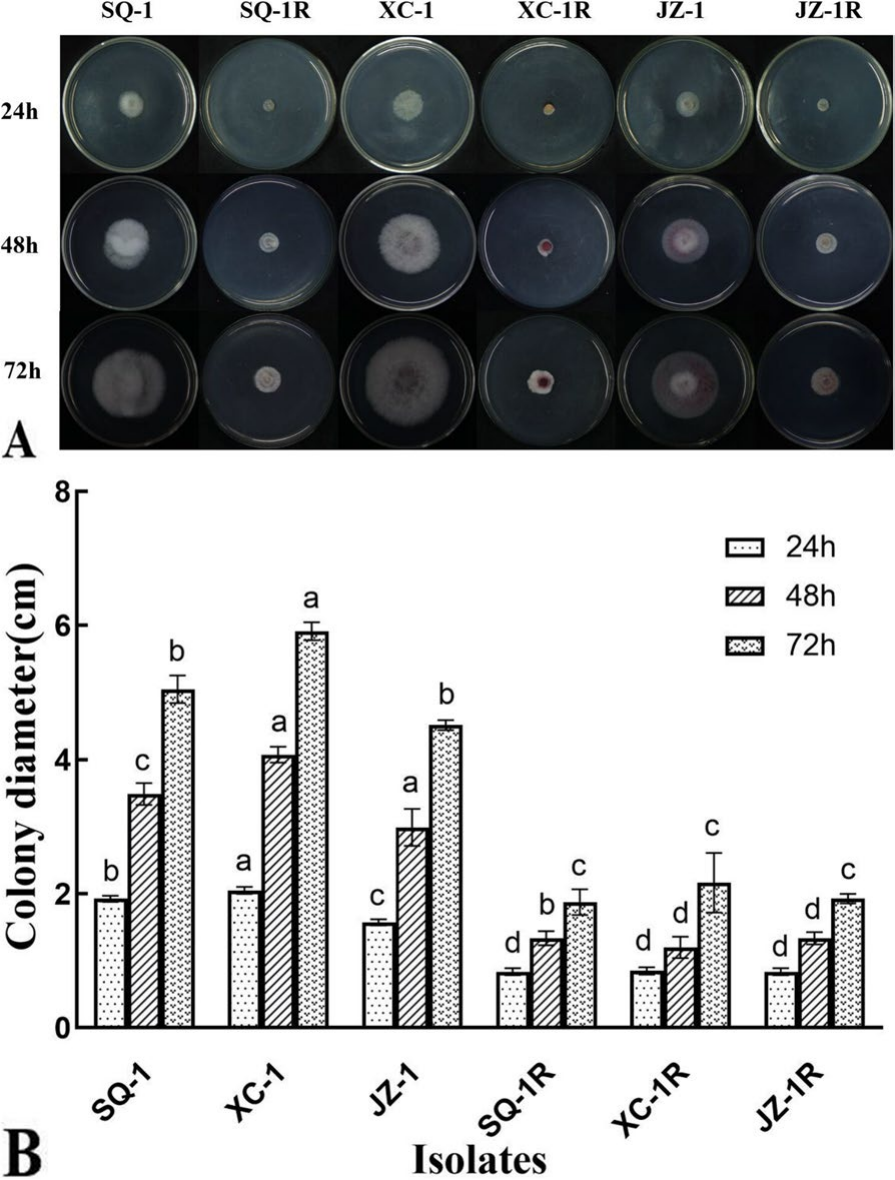
**A-B** Mycelial growth of three prothioconazole-resistant mutants of *F. pseudograminearum.* The average colony diameter of three resistant mutants (SQ-1R, XC-1R, and JZ-1R) and their wild-type parental isolates (SQ-1, XC-1, and JZ-1) was measured after incubation on PDA at 25 °C for 24, 48, and 72 h. Data represent the mean of six replicates ± standard error (SE). Different letters above columns indicate significant differences according to Fisher’s least significant difference test (*p* < 0.05)

**Fig. 2:**
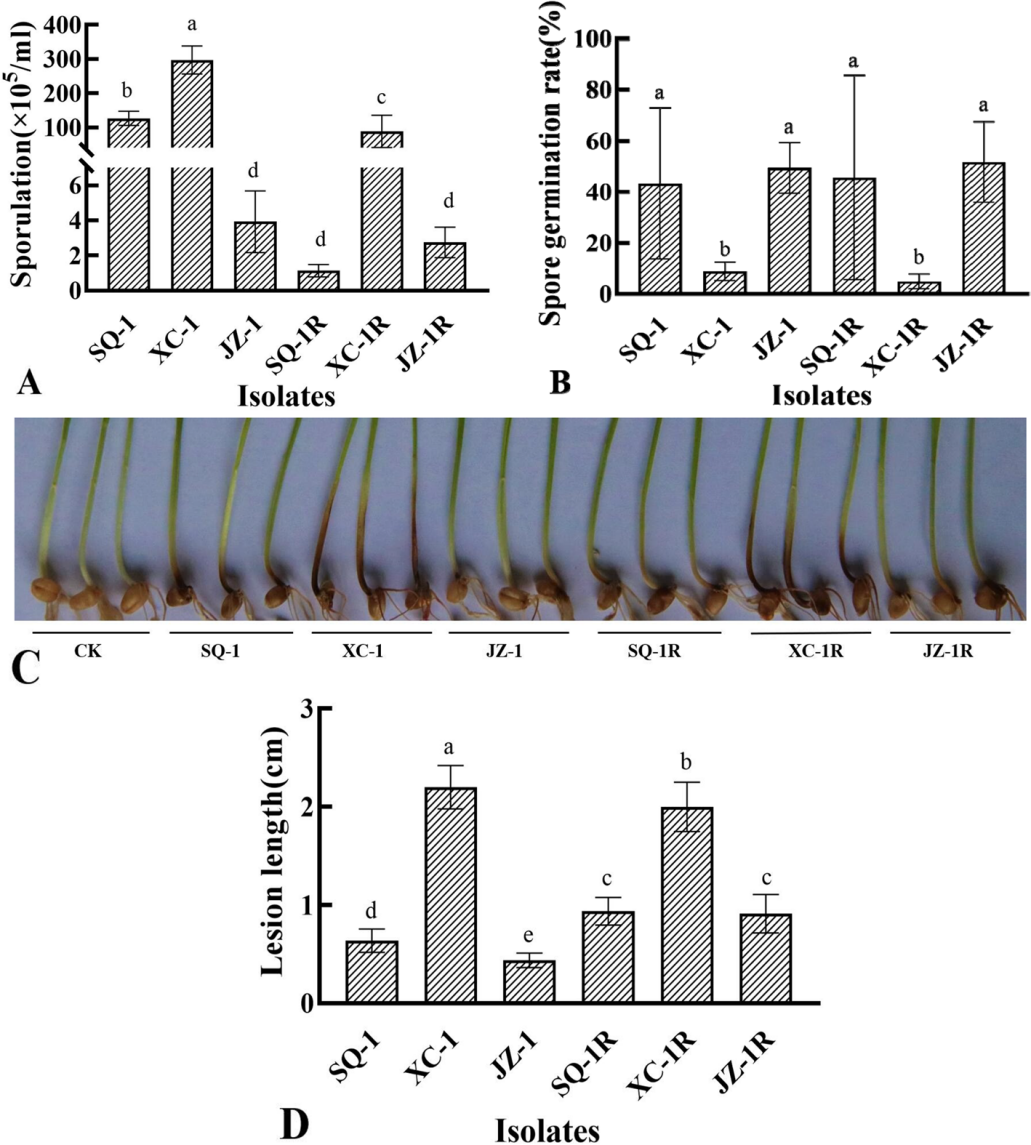
**A-D** Sporulation, germination rate, disease symptoms and pathogenicity of three prothioconazole-resistant mutants of *F. pseudograminearum.* The sporulation and germination rate of three resistant mutants and their wild-type parental isolates after incubation for 3 days in mung bean broth at 25 °C with shaking (130 rpm). Data represent the mean of six replicates ± SE. Disease symptoms caused by three prothioconazole-resistant mutants of *F. pseudograminearum* in comparison to their parental isolates when assessed on wheat seedlings at 14 days post-inoculation (dpi), as well as pathogenicity data based on lesion size. Data represents the mean of 10 coleoptiles and two independent experiments. Different letters above the columns indicate significant differences according to Fisher’s least significant difference test (*p* < 0.05)

The mycelial growth of the prothioconazole-resistant mutants (SQ-1R, XC-1R, and JZ-1R) was dramatically reduced compared to the wild-type parental isolates, and differed significantly (*p* < 0.05) even at 24 h post inoculation (hpi), although the difference was more evident at 48 and 72 hpi (Fig. 1). The sporulation of the mutants was also affected, with all of the mutants producing fewer spores than their parental isolates (Fig. 2A), although the difference was only significant in two of prothioconazole-resistant mutants (XC-1R and SQ-1R). Although, spore production was reduced by as much as 90% in the most affected mutant (SQ-1R), the germination rate of the spores that were produced by the mutants, did not significantly differ from those of the parental isolates (Fig. 2B). However, all of the prothioconazole**-**resistant mutants were found to have significantly (*p* < 0.05) altered pathogenicity in wheat coleoptiles (Fig. 2C, D), with the size of the lesions being greater in two of the mutants (SQ-1R and JZ-1R), and reduced in the third (XC-1R). Taken together, these results demonstrate that there is some fitness cost associated with prothioconazole resistance, especially with regard to mycelial growth, but that the biological characteristics associated with prothioconazole resistance can vary, and even result in increased pathogenicity.

### Sequence analysis of the *FpCYP51A*, *FpCYP51B*, and *FpCYP51C* genes in prothioconazole-resistant mutants of *F. pseudograminearum*

The DNA sequences of the three members of the*CYP51* subfamily genes cloned from the *F. pseudograminearum* isolates (FPSE_00109, FPSE_01496, and FPSE_02459 genes which homologous to the *FgCYP51A, FgCYP51B,* and *FgCYP51C* in *F. graminearum*, respectively) were found to be highly homologous to the corresponding genes from the closely related species *Fusarium graminearum* (data not shown), including *FgCYP51A* (FGSG_04092)*, FgCYP51B* (FGSG_01000) and *FgCYP51C* (FGSG_11024). Further investigation, comparing the predicted amino acid sequences of the various CYP subfamily genes from the resistant mutants with those of their parental isolates, identified several amino acid changes that might be associated with prothioconazole resistance (Table 2). Changes were detected in all of the CYP paralogues, but perhaps most notable were three conserved amino acid substitutions (M63I, A205S, and I246V) that occurred in the *FpCYP51C* gene of all three of the mutants. A fourth substitution (L298Q) was also found in the *FpCYP51C* sequence of XC-1R. In addition, multiple non-conserved substitutions were detected in the other two genes. N216D, S376P and S388P were found in the *FpCYP51A* gene of SQ-1R as well as D481V in the *FpCYP51A* gene of XC-1R. K438T and F182C were found in the *FpCYP51B* gene of SQ-1R and JZ-1R, respectively, and two substitutions, F90T and I167V, in the *FpCYP51B* gene of XC-1R. Taken together these results suggest that the three conserved mutations in the *FpCYP51C* sequence were most likely the cause of the observed prothioconazole resistance of the mutants, but that the occurrence of the non-conserved substitutions perhaps could explain the differences in the biological characteristics of the three resistant mutants.

**Table 2:** Amino acid substitutions occurring in the predicted sequences of three members of *CYP51* subfamily genes in prothioconazole-resistant mutants of *F. pseudograminearum*

Subunit	Mutant	Amino acid substitution	Reference
FpCYP51A	SQ-1R-1	N216D, S376P, S388P	Current study
	XC-1R-1	D481V	Current study
	JZ-1R-1	/	Current study
	HB2107R1, ZK2105R1, and AY2109-R3	/	[6]
FpCYP51B	SQ-1R-2	K438T	Current study
	XC-1R-2	F90T, I167V	Current study
	JZ-1R-2	F182C	Current study
	HB2107R1, ZK2105R1, and AY2109-R3	/	[6]
FpCYP51C	SQ-1R-3	M63I, A205S, I246V	Current study
	XC-1R-3	M63I, A205S, I246V, L298Q	Current study
	JZ-1R-3	M63I, A205S, I246V	Current study

/ Indicates no amino acid mutation available

### Relative expression of the *FpCYP51A*, *FpCYP51B*, and *FpCYP51C* genes in prothioconazole-resistant mutants of *F. pseudograminearum*

**Fig. 3:**
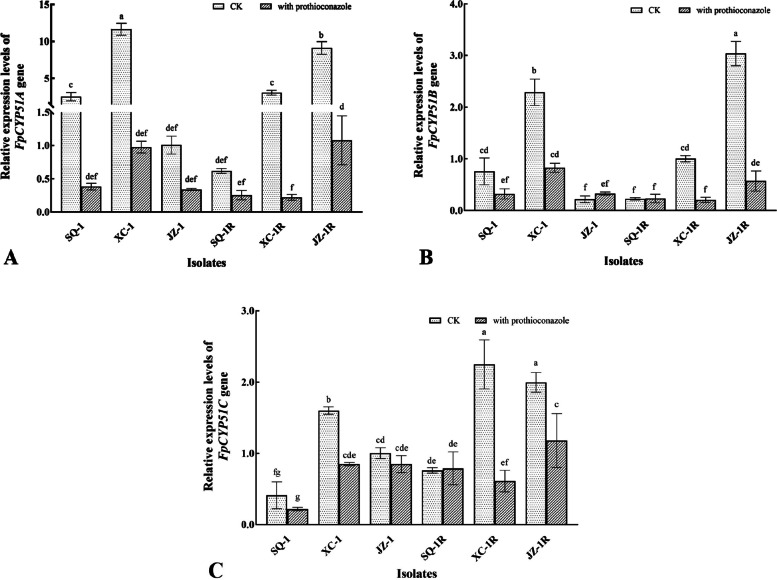
**A-C** Relative expression of three *FpCYP51* genes in prothioconazole-resistant mutants of *F. pseudograminearum.* The relative expression of three *FpCYP51* genes in both the resistant mutants and wild-type parental isolates was compared in the absence and presence of prothioconazole (0.1 μg/mL), with β-tubulin as the reference gene. Different letters above the columns indicate significant differences according to Fisher’s least significant difference test (*p* < 0.05). Bars indicate standard error (SE)

The qPCR analysis conducted in the current study found that in general the presence of prothioconazole (0.1 μg/mL) dramatically reduced the expression of the three *FpCYP51* genes, in both the resistant mutants as well as the sensitive parental isolates (Fig. 3). The two exceptions were the wild-type strain, JZ-1, which exhibited a slight increase in the expression of FgCYP51B, and the SQ-1R mutant, in which the presence of the fungicide had no significant (*p* < 0.05) effect on either *FpCYP51B* or *FpCYP51C*, although it did increase expression of *FpCYP51A*. The analysis also identified altered expression when comparing the mutants to the wild-type parental isolates. For example, both SQ-1R and XC-1R exhibited significantly (*p* < 0.05) reduced expression of their *FpCYP51A* and *FpCYP51B* genes in comparison to SQ-1 and XC-1, but increased expression of *FpCYP51C*, although the expression of *FpCYP51C* in the presence of the fungicide was significantly (*p* < 0.05) reduced in XC-1R compared with XC-1. In contrast, JZ-1R exhibited a more uniform pattern of expression that differed to the other two mutants in that its three *FpCYP51* genes were always significantly (*p* < 0.05) overexpressed in comparison to its parental isolate, JZ-1. However, it was interesting to note that although all of the wild-type parental isolates were similar in their general pattern of expression i.e. reduced expression in response to prothioconazole, absolute levels of expression exhibited a high degree of variation, both between isolates, as well as for the different *FpCYP51* genes. Taken together, these results confirm that the expression of the three *FpCYP51* genes was effected by both the presence of the fungicide, and by prothioconazole resistance (likely caused by the three conserved substitutions), but that the non-conserved mutations, or wider genetic differences among the three different parental isolates might also affect expression within a complex interaction.

### Cross-resistance between prothioconazole and other fungicides

The results of the current study found no evidence of cross-resistance between prothioconazole and any of the other fungicides assessed, including tebuconazole, prochloraz, carbendazim, flutriafol, pyraclostrobin, fluazinam, and fludioxonil (Table 3). This result indicates that in addition to providing effective control of *F. pseudograminearum*, prothioconazole could help to prevent the risk of resistance emerging to these fungicides by taking advantage of differences in their modes of action.

**Table 3:** Cross-resistance between prothioconazole and other commonly used fungicides

Fungicides	Sensitive parental isolates (EC_50_, μg/mL)			Prothioconazole-resistant mutants (EC_50_, μg/mL)		
	**SQ-1**	**XC-1**	**JZ-1**	**SQ-1R**	**XC-1R**	**JZ-1R**
Prothioconazole	0.933	1.066	1.342	14.809	12.590	21.030
Tebuconazole	0.055	0.049	0.09	0.072	0.049	0.021
Prochloraz	0.01	0.005	0.007	0.008	0.005	0.005
Carbendazim	0.199	0.197	0.231	0.458	0.119	0.279
Flutriafol	0.168	0.512	0.54	0.951	0.647	0.115
Pyraclostrobin	0.124	0.053	0.199	0.359	0.047	5.395
Fluazinam	0.014	0.015	0.018	0.015	0.02	0.009
Fludioxonil	0.021	< 0.003125	< 0.003125	< 0.003125	< 0.003125	< 0.003125

## Discussion

### Prothioconazole resistance not observed in field isolates of *F. pseudograminearum*

*Fusarium pseudograminearum* is a soil-borne pathogen that is the most common causal agent of FCR, a highly destructive disease that constrains wheat production in China. Indeed, FCR has become an increasing concern in Huanghuai region, the key wheat growing region of China in recent years, and the control of FCR is limited to the use of chemical fungicides in the absent of resistant varieties [5]. However, to date, no chemicals have been registered specifically for the control of FCR in China [5, 6]. The current study, confirmed previous findings that prothioconazole, a triazole DMI fungicide developed by Bayer Crop Protection Company, exhibits high activity against *F. pseudograminearum* [6], with all of the 67 field isolates surveyed being found incapable of growth on PDA amended with 5.0 μg/mL prothioconazole (Table 1). The EC_50_ values of the isolates ranged from 0.016 to 2.974 μg/mL, with an average of 1.191 ± 0.72 μg/mL (mean ± SD), which was similar to the results of Wei et al. [6], and further evidence that all of the isolates were susceptible to prothioconazole and that no field resistance had emerged.

### Mycelial growth, sporulation and pathogenicity of prothioconazole-resistant mutants of *F. pseudograminearum*

Repeated exposure to the fungicide under laboratory conditions resulted in the production of three highly resistant *F. pseudograminearum* mutants (SQ-1R, XC-1R, and JZ-1R) that provided the opportunity to investigate the potential risks of prothioconazole resistance. Although the mutants had significantly higher EC_50_ values (14.809, 12.590 and 21.030 μg/mL), it was found that resistance was accompanied by a certain fitness cost that resulted in significantly impaired mycelial growth, a finding that was in agreement with the previous study of Wei et al. [6]. The study also found that two of the mutants exhibited much lower rates of sporulation, although the germination of the spores that were produced was not found to differ significantly to those of the parental isolates in any of the mutants tested. However, in contrast to the previous study [6], it was found that two of the mutants exhibited increased pathogenicity in wheat coleoptiles (Fig. 2C, D). The exception is which the third (XC-1R) was less pathogenic than its parental isolate (XC-1), which was the most virulent of the three wild-type isolates tested, it was still more pathogenic than the other two wild-type isolates (SQ-1 and JZ-1). These results indicate that although resistant mutants might not be able to spread as easily as wild-type isolates, once they form an infection they are at least as damaging as wild-type isolates. However, it should also be noted that the current study evaluated pathogenicity by artificially inoculated wound sites, and that the mutants might still be impaired in their capacity to form natural infections. Further investigation is therefore necessary to establish the potential risk of prothioconazole resistance under field conditions.

### Sequence analysis and relative expression of the *FpCYP51A*, *FpCYP51B*, and *FpCYP51C* genes in prothioconazole-resistant mutants of *F. pseudograminearum*

Previous studies have shown that the *FgCYP51A*, *FgCYP51B,* and *FgCYP51C* genes play a key role in the pathogenicity and DMI sensitivity of *F. graminearum*, which is a species closely related to *F. pseudograminearum*. For example, it was found that knock-out mutants lacking the *FgCYP51A* gene exhibited increased sensitivity to triazole fungicides [19], and that although FgCYP51B seems to play a key role as a sterol 14α-demethylase, a functional *FgCYP51A* gene could rescue mutants lacking *FgCYP51B*. Meanwhile, *FgCYP51C* is important in the synthesis of the deoxynivalenol (DON) toxin, and thus the pathogenicity of *F. graminearum* in wheat [20, 21]. It was therefore not surprising to find an association between prothioconazole, the three *FpCYP51* homologues, and the wider biology of *F. pseudograminearum*. In this case it was found that three conserved mutations (M63I, A205S, and I246V) in the predicted *FpCYP51C* sequence were likely the cause of the observed resistance of three different *F. pseudograminearum* mutants (Table 2). In addition, there were several non-conserved substitutions in the *FpCYP51A* and *FpCYP51B* genes, none of which had been observed in a previous study of prothioconazole resistance in *F. pseudograminearum* [6]. Further research, including site directed mutagenesis is required not only to ascertain the precise contribution that each mutation might play in the prothioconazole resistance observed in the current study, but also to investigate the causes of the altered gene expression and biological characteristics of the three prothioconazole**-**resistant mutants.

### Cross-resistance between prothioconazole and other fungicides

The current study found no evidence of cross-resistance between prothioconazole and any of the other fungicides assessed, including prochloraz, flutriafol, and the triazole tebuconazole, which are all DMI fungicides (Table 3). This result was particularly encouraging as it is well known that the long-term use of a single fungicide can result in the emergence of fungicide resistance, but that the use of different chemicals either in combination, or in rotation can delay this process [8]. Of course, this result may also be due to the low resistance multiple of the tested resistant, prothioconazole resistant mutants, SQ-1R, XC-1R, and JZ-1R had EC_50_ values of 14.809, 12.590, and 21.030 μg/mL, respectively (Table 3). Indeed, market leaders including Bayer, Syngenta and BASF, have already developed formulations of prothioconazole in combination with SDHI fungicides such as penflufen and fluopyram, or strobilurins such as fluoxastrobin and trifloxystrobin for the treatment of many plant diseases including powdery mildew, rust disease, downy mildew and so on. In addition to delaying the development of fungicide resistance, it has also been noted that fungicide combinations can result in synergistic effects that improve their effectiveness. For example, a previous study found that seed dressing that combined phenamacril with difenoconazole or fludioxonil provided enhanced control of FCR [22]. Further research is required to optimize such formulations, but the results of the current study indicate that the combination of prothioconazole with other fungicides has great potential to provide effective and long-lasting control of FCR, and thereby sustain high yields in the wheat fields of China.

## Conclusions

No prothioconazole-resistant isolates of *F. pseudograminearum* were found in the field, and the resistant isolates obtained by continuously exposed to prothioconazole in the laboratory showed lower fitness compared to parental isolates in this study. What’s more, we found the new insight into the resistant mechanism and biological characteristics associated with prothioconazole resistance in *F. pseudograminearum*, but also strong evidence that prothioconazole could provide effective and sustained control of FCR, especially when applied in combination with other fungicides. Taken together, this study evaluated the resistance level of *F. pseudograminearum* to prothioconazole as a means to assess the safety of prothioconazole and to provide a theoretical basis for future control strategies against FCR.

### Methods

## Experimental materials and fungicides

A total of 67 *F. pseudograminearum* isolates were surveyed from various locations in the wheat growing regions which exhibiting typical symptoms of FCR in the field of Henan Province of China between 2019 and 2021 as detailed in Table 1. The candidate isolates were isolated and purified by conventional culture methods, and further identified by molecular biology of their Internally Transcribed Spacer (ITS), as well as pathogenicity experiments to confirm the severity of FCR symptoms in the wheat seeding (Data not shown).The fungal isolates collected were routinely cultured on potato dextrose agar (PDA; potato 200.0 g/L, agar 20.0 g/L, dextrose 20.0 g/L), with spore samples being suspended in 20% glycerin solution for long-term storage at -20°C.

Three prothioconazole-sensitive isolates, including XC-1, SQ-1 and JZ-1, with 50% effective inhibitory concentrations (EC_50_) of 0.933, 1.066 and 1.342 μg/mL, respectively, were selected for the production of prothioconazole-resistant mutants by repeated exposure to prothioconazole under laboratory conditions according to the previous study [23, 24]. Brieflfly, each mutant was subjected to 10 successive rounds of subculture on prothioconazole-free PDA before their EC_50_ values were re-evaluated on PDA containing prothioconazole at the following concentrations: 0, 1.0, 2.0, 4.0, 8.0, 16.0, 32.0, 64.0, 128.0, 256, and 300.0 µg/mL. The resulting mutants, XC-1R, SQ-1R and JZ-1R, were highly resistant to prothioconazole, with EC_50_ values of 14.809, 12.590 and 21.030 μg/mL, respectively.

Most of the technical-grade fungicides used in the current study, including 97.0% prothioconazole (Kangbaotai Fine-Chemical Co. Ltd.), 96.2% tebuconazole (Sheyang Huanghai Pesticide Chemical Co. Ltd.), 95% prochloraz (Kangbaotai Fine-Chemical Co. Ltd.), 95.3% flutriafol (Guangxi Tianyuan Biochemical Co., Ltd), 97.5% pyraclostrobin (Kangbaotai Fine-Chemical Co. Ltd.), 96.0% fluazinam (Hubei Jianyuan Chemical Co. Ltd.), and 96.0% fludioxonil (Hubei Jianyuan Chemical Co. Ltd.), were dissolved in acetone (Analytically pure, Tianjin Fuyu Fine Chemical Co., Ltd) to prepare 10,000 µg/mL stock solutions. The one exception was the 98.1% carbendazim (Haili Guixi Chemical Co., Ltd.), which was dissolved in 0.1 mol/L hydrochloric acid (HCl, Analytically pure, Tianjin Fuyu Fine Chemical Co., Ltd). The resulting stock solutions were stored at 4°C for no more than 2 weeks before being used to prepare the serial dilutions used in the experiments. Mycelial growth assays were performed to ensure that the solvents had no effect on the growth of *F. pseudograminearum* at the concentrations used (data not shown).

### Prothioconazole sensitivity assay

The mycelial growth assay described in a previous study [23] was adapted to determine both the baseline sensitivity for prothioconazole in *F. pseudograminearum*, as well as to confirm the resistance status of individual isolates or mutants. Briefly, mycelial plugs (5 mm in diameter) were cut from the margins of 48-h-old colonies and transferred to fresh PDA containing various concentrations of prothioconazole (0, 0.00625, 0.0125, 0.025, 0.05, 0.1, 0.2, 0.4, 0.8, 1.6 and 3.2 μg/mL) when determining the baseline sensitivity, and 5.0 µg/mL as an discriminatory dose. Growth was assessed after 48 h of incubation at 25°C by measuring the colony diameter in two perpendicular directions, while the ability to grow at 5.0 µg/mL was considered the threshold to classify isolates as resistant. The data collected was used to construct inhibition curves and determine EC_50_ values, while the percentage of resistant isolates was calculated as follows: percentage of resistant isolates (%) = total number of resistant isolates/total number of isolates detected × 100.

### Biological characteristics of prothioconazole-resistant *F. pseudograminearum* mutants

#### Mycelial growth

The mycelial growth assay described above was used to assess the growth of three prothioconazole-resistant mutants (XC-1R, SQ-1R and JZ-1R) in comparison to their parental isolates (XC-1, SQ-1 and JZ-1). In this case, these isolates/mutants were cultured on PDA plates in the absence of prothioconazole at 25 ℃ and in dark for 24, 48 and 72 h, respectively, and the colony diameters were measured to compare the mycelial growth rates of the the parental isolates and resistant mutants. Each isolate/mutant was represented by six replicate plates, and the whole experiment performed once.

#### Sporulation

The sporulation of the three resistant mutants was compared to that of the parental isolates using mung bean broth as described in a previous study [25]. The test colonies were initially established by transferring 5 mm mycelial plugs from 2-day-old PDA cultures to flasks containing 30 mL mung bean broth (MBB). After 3 days of incubation at 23 ℃ with shaking(130 rpm), the resulting spores were harvested and counted using a hemocytometer (Shanghai Qiujing Biochemical Reagent Instrument Co. Ltd.). Each mutant/isolate was represented by at least 3 independent replicates, and the whole experiment performed once.

#### Pathogenicity on wheat

The pathogenicity of the prothioconazole-resistant mutants was assessed on wheat seedlings as described previously with slight modifications [23, 24]. Coleoptiles of the wheat cultivar Bainong 207 (provided by wheat engineering center of henan institute of science and technology) were inoculated after 3 days of growth by cutting them open and applying 5 μL spore suspension (1 × 10^5^ spores/mL) to the exposed surface. Positive and negative controls were prepared in an identical manner using the parental isolates and sterile water, respectively. The seedlings were then incubated at 23°C with 95% relative humidity and a 16 h photoperiod for 15 days, at which point the length of the infection lesions was measured. Each mutant/isolate was represented by 10 separate coleoptiles, and the entire experiment performed twice.

#### Cloning and sequencing of the *FpCYP51A*, *FpCYP51B*, and *FpCYP51C* genes

Fresh mycelium was collected from 200 mL cultures grown in potato dextrose broth (PDB) medium, and the genomic DNA extracted according to the method of a previous study [26]. The full-length sequence of each gene was then amplified using PCR and the following sequence-specific primer sets: FPSE_00109_F/FPSE_00109_R, TCACAACCAATACCCATTCAACC/CTTGACGCTCAGTCTCTATTGAC; FPSE_01496_F/FPSE_01496_R,

ATGGGTCTCCTTCAAGAAC/TTACTGGCGTCGCTCCCAG; FPSE_02459_F/FPSE_02459_R, ATGGACTCTCTCTATGAGAC/TCATTCTACTGTCTCGCGTC, which were designed using the method detailed in a previous study [27]. The PCR itself was performed using a 50.0 μL reaction mix containing 25.0 μL of 2.0 × ES Taq Master Mix (CoWin Biosciences), 1.5 μL of template DNA, 2.0 μL of each primer and 21.5 μL of ultrapure water, and processed using a 96-well thermal cycler (Applied Biosystems, Thermo Fisher Scientific) with the following program: initial denaturation at 94°C for 5 min; followed by 35 cycles of 94°C for 30 s, 57°C for 30 s, and 72°C for 1.5 min; and a final extension at 72°C for 10 min. The resulting PCR products were then purified, and cloned into the PMD_19-T vector using a commercial cloning kit (TaKaRa) before being sequenced (Wuhan Gene-create Biotechnology Co. Ltd). The sequencing data obtained were analyzed using DNAMAN software (Version 6.0; Lynnon Biosoft), and the predicted amino acid sequences compared to identify amino acid differences between the resistant mutants and the sensitive parental isolates as described previously [25].

#### qRT-PCR analysis of *FpCYP51A*, *FpCYP51B*, and *FpCYP51C* expression

Total RNA was extracted from mycelial samples of both the resistant mutants and parental isolates, which had been grown in either the absence or presence prothioconazole (0.1 μg/mL), using a fungal RNA kit (Omega Bio-Tek) following the protocol of the manufacturer. First-strand cDNA was prepared using the PrimeScript RT kit (TaKaRa), and partial sequences of the *FpCYP51A, FpCYP51B,* and *FpCYP51C* genes, as well as the β-tubulin reference gene amplified using the following primer sets: FPSE_00109qPCR_F/FPSE_00109qPCR_R, GCTTACGGCCTGGACCCTTG/TCTTCGGCGTTGGCATCCTG; FPSE_01496qPCR_F/FPSE_01496qPCR_R, GGTGTCAGCGCTACCGACTC/GCCCTTGCTGACAAGACCGT; FPSE_02459qPCR_F/FPSE_02459qPCR_R TGCGGACTCTACCGCTCTCA/GGTGGACGATGCGGGTTAGG, and Fgβ-tubulin_F/Fgβ-tubulin_R,

GATGGCTGCCTCCGACTTCC/ CGCAGAGAGCGGTCTGGATG, respectively. The quantitative real-time PCR (qRT-PCR) itself was performed using the Quantstudio 6 Flex PCR Detection System (Thermo Fisher) with SYBR Green I fluorescent dye. The relative expression was calculated using the method described previously [24] using β-tubulin as the reference gene [28]. Each gene and mutant/isolate combination was represented by three biological replicates, which were then used to calculate the mean and standard error (SE).

#### Cross-resistance between prothioconazole and other fungicides

The potential for cross-resistance between prothioconazole and eight commonly used fungicides, including tebuconazole, prochloraz, carbendazim, flutriafol, pyraclostrobin, fluazinam, and fludioxonil, was assessed using the mycelial growth assay described above. The EC_50_ values for each fungicide in both the prothioconazole-resistant mutants and sensitive parental isolate were assessed on PDA amended with a range of fungicide concentrations: 0.05, 0.1, 0.2, 0.4, 0.8, 1.6, 3.2, and 6.4 μg/mL for flutriafol, pyraclostrobin, and carbendazim, respectively; 0.025, 0.05, 0.1, 0.2, 0.4, 0.8, 1.6, and 3.2 μg/mL for tebuconazole; 0.003125, 0.0625, 0.0125, 0.025, 0.05, 0.1, 0.2, and 0.4 μg/mL for prochloraz; 0.0625, 0.0125, 0.025, 0.05, 0.1, 0.2, 0.4, and 0.8 μg/mL for fludioxonil and fluazinam; and 0.1, 0.4, 0.8, 1.6, 3.2, 6.4, 12.8, and 25.6 μg/mL for prothioconazole. At least three replicate plates were prepared for each fungicide mutant/isolate combination, and the entire experiment performed three times.

### Statistical analyses

The data collected in the current study were initially analyzed by ANOVA using SPSS software (Ver. 17.0; SPSS Inc.), while statistical differences were determined using Fisher's least significant difference test (*α* = 0.05).

### Supplementary Information

**Additional file 1: Supplementary Fig 1.** Frequency distribution of prothioconazole sensitivity among 67 F. pseudograminearum isolates.

## Data Availability

All data generated or analyzed in this study are available from the corresponding author on reasonable request. The data during the current study are available from the NCBI Nucleotide database under accession numbers CM003199, CM003198 and CM003200.
