# Supplementary material for: Survey of prothioconazole sensitivity in Fusarium pseudograminearum isolates from Henan Province, China, and characterization of resistant laboratory mutants
Source: BMC Plant Biol. 2024 Jan 4;24:29. doi: 10.1186/s12870-023-04714-w (PMC10765739; doi:10.1186/s12870-023-04714-w)
Supplement: Supplementary file 1 — Additional file 1: Supplementary Fig 1. Frequency distribution of prothioconazole sensitivity among 67 F. pseudograminearum isolates. [file 12870_2023_4714_MOESM1_ESM.pdf]

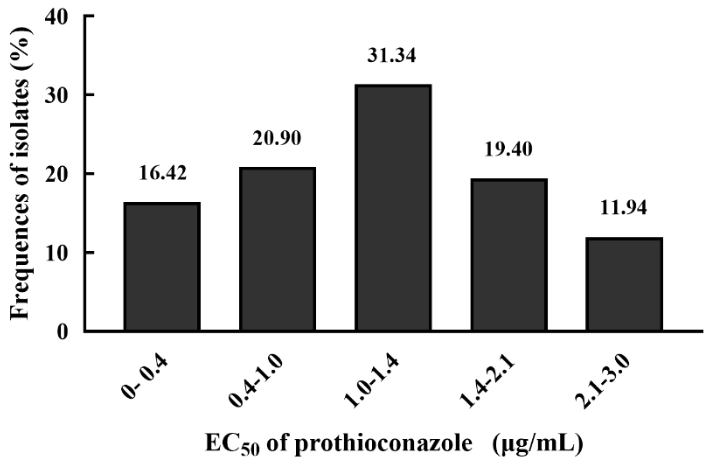

Supplementary Fig. 1 Frequency distribution of prothioconazole sensitivity among 67 *F. pseudograminearum* isolates
